# Striatal Cholinergic Interneurons: How to Elucidate Their Function in Health and Disease

**DOI:** 10.3389/fphar.2019.01488

**Published:** 2019-12-13

**Authors:** Nicolas Mallet, Arthur Leblois, Nicolas Maurice, Corinne Beurrier

**Affiliations:** ^1^ Université de Bordeaux, Institut des Maladies Neurodégénératives, Bordeaux, France; ^2^ CNRS UMR 5293, Institut des Maladies Neurodégénératives, Bordeaux, France; ^3^ Aix Marseille Univ, CNRS, IBDM, Marseille, France

**Keywords:** striatal cholinergic interneurons, basal ganglia, optogenetics, computational modelling, Parkinson’s disease

## Abstract

Striatal cholinergic interneurons (CINs) are the main source of acetylcholine in the striatum and are believed to play an important role in basal ganglia physiology and pathophysiology. The role of CINs in striatal function is known mostly from extracellular recordings of tonically active striatal neurons in monkeys, which are believed to correspond to CINs. Because these neurons transiently respond to motivationally cues with brief pauses, flanked by bursts of increased activity, they are classically viewed as key players in reward-related learning. However, CIN modulatory function within the striatal network has been mainly inferred from the action of acetylcholine agonists/antagonists or through CIN activation. These manipulations are far from recapitulating CIN activity in response to behaviorally-relevant stimuli. New technical tools such as optogenetics allow researchers to specifically manipulate this sparse neuronal population and to mimic their typical pause response. For example, it is now possible to investigate how short inhibition of CIN activity shapes striatal properties. Here, we review the most recent literature and show how these new techniques have brought considerable insights into the functional role of CINs in normal and pathological states, raising several interesting and novel questions. To continue moving forward, it is crucial to determine in detail CIN activity changes during behavior, particularly in rodents. We will also discuss how computational approaches combined with optogenetics will contribute to further our understanding of the CIN role in striatal circuits.

## Introduction

The striatum is a brain region containing high levels of acetylcholine (ACh), muscarinic receptors, and other ACh-related markers (Weiner et al., 1990; Hersch et al., 1994). Cholinergic interneurons (CINs) are the main source of ACh in the striatum [but see (Dautan, 2014)]. Despite their small numbers (1–3% of all striatal cells) and scattered distribution throughout the striatum, CINs have dense terminal fields that overlap those of dopaminergic projections coming from the substantia nigra pars compacta (Bolam et al., 1984). CINs contact the two populations of striatal output neurons (also called medium spiny neurons, MSNs) that express either the dopamine D1 or D2 receptors. While most striatal neurons are not autonomously active, CINs exhibit a regular spiking activity in absence of any synaptic inputs (Bennett et al., 2000). Extracellular recordings performed *in vivo* in the striatum of monkeys also reveal the presence of tonically active neurons (TANs), which are thought to correspond to CINs (Aosaki et al., 1995). Hence, the morphofunctional features of CINs—mainly their extensive arborization primarily directed to MSNs and their tonic activity—place them as potent modulators of striatal output. Striatal output regulation is a fundamental process of the basal ganglia functioning, as a balanced activity between D1 and D2 MSNs is required to ensure correct motor and cognitive behaviors.

The improvement of parkinsonian tremor by both dopaminergic agonists and anticholinergic drugs led to the dopamine (DA)-ACh balance hypothesis, where DA and ACh are believed to play opposite roles in the striatum (Barbeau, 1962). Even though the prescription of anticholinergic drugs has been phased out due to their side-effects, this long standing clinical observation underlines the functional impact of ACh as the level of DA falls and has often led to the consideration of Parkinson’s disease (PD) as a hypercholinergic disorder [but see (McKinley, 2019)]. There is indeed compelling evidence showing that DA depletion triggers complex alterations in striatal cholinergic signaling, activity, and connectivity (Aosaki, 1994; Raz, 2001; Ding, 2006; Salin, 2009). However, there is no consensual view explaining how CINs contribute to motor symptoms and abnormal network dynamic in PD.

At the cellular level, CIN modulation of the striatal network has been mainly inferred from the action of ACh agonists or through CIN activation. While nicotinic receptors (nAChRs) are expressed by interneurons and extrinsic afferent terminals, MSNs respond to ACh exclusively *via* muscarinic receptors (mAChRs): M1 receptors are present on D1 and D2 MSNs and M4 receptors are preferentially expressed by D1 MSNs. Activation of mAChRs modulates an array of voltage-gated channels and intracellular pathways in MSNs. Determining the combinatorial effect of these actions, potentially even opposing each other, is highly challenging and has recently been covered at length by excellent recent reviews (Tanimura, 2018; Ztaou and Amalric, 2019; Abudukeyoumu et al., 2019). A hallmark of CINs is their continuous tonic activity, which is expected to lead to a high level of ACh in the striatum, and the stereotypical bursts and pauses activity that they acquire during sensorimotor learning (Apicella, 2007). We can assume that a drop in ACh release, as expected to happen after a brief decrease in firing, conveys meaningful information to the striatal network. A recent hypothesis proposes that the pause would open a permissive temporal window during which corticostriatal synaptic plasticity occurs (Deffains and Bergman, 2015). However, it is still unclear how inhibition of CIN activity shapes striatal properties. Here, we review the related literature and show how optogenetic and computational approaches may contribute to further our understanding of this topic.

## Consequences of Cholinergic Interneuron Inhibition on Striatal Properties

The widespread excitatory input from the cortex targeting D1 and D2 MSNs sets the activity of the direct and indirect striatofugal pathways which play a fundamental role in movement planning and action selection. Understanding how CINs modulate the dynamics of corticostriatal processing and MSN activity is therefore essential to uncover basal ganglia function. Cholinergic modulation of long-term corticostriatal plasticity has been addressed in excellent reviews (Lovinger, 2010; Lerner and Kreitzer, 2011) and will not be further discussed here.

The effects of cholinergic antagonists on corticostriatal transmission might provide interesting insights to predict how a pause in CIN firing impacts striatal output. It was reported that atropine, a broad mAChRs antagonist, or methoctramine, at a concentration that blocks M2 and M3 mAChRs, lead to a modest increase in corticostriatal transmission *via* the inhibition of mAChRs located on the glutamatergic terminals, suggesting the existence of tonic cholinergic presynaptic inhibition (Pakhotin and Bracci, 2007). On the other hand, pirenzepine, a blocker of M1 mAChRs, reduces corticostriatal transmission (Wang, 2006; Tozzi, 2011). In these last two studies, the authors suggest that lowering M1 mAChR activity in MSNs leads to the opening of L-type Ca^2+^ channels, which then triggers endocannabinoids release. Endocannabinoids are then able to reduce glutamate transmission by activating presynaptic CB1 receptors. Hence, mAChRs inhibition could exert opposite actions on basal corticostriatal transmission depending on their pre- or postsynaptic localization. Nicotinic α7 receptors have been described on cortical glutamatergic terminals but whether these receptors directly modulate corticostriatal transmission is still unclear (Howe et al., 2016).

One of the caveats of pharmacological experiments is that they do not allow to assess the effects of endogenous ACh that depends on the temporal dynamic of CIN firing. Moreover, CINs might co-release glutamate and GABA along with ACh, with effects that cannot be apprehended through this approach (Higley et al., 2009; Lozovaya, 2018). Optogenetic manipulations, enabling control of electrical activity in specific cell types with high temporal accuracy, can provide substantial insights into these issues. Here, we review the few studies showing the impact of optogenetic inhibition of CIN activity in the dorsal striatum. *In vitro*, we and others have shown that inhibition of CIN firing with halorhodopsin (eNpHR) is associated with a decrease in D1 and D2 MSN excitability that might involve a lowering of M1 mAChRs activation (Maurice, 2015; Zucca et al., 2018). In anaesthetized mice, opto-inhibition of eNpHR-expressing CINs was also reported to decrease MSN activity by hyperpolarizing their membrane potential and increasing the duration of down states (Zucca et al., 2018). In contrast, eNpHR-induced inhibition of CIN firing in freely moving mice did not alter MSN activity (English, 2011). In this last study, it is the rebound of action potentials occurring at the end of the eNpHR-induced hyperpolarization that triggered a decrease in MSN firing. Interestingly, despite the cellular effect induced by this pause-rebound, it was not followed by any detectable behavioral responses (English, 2011). This is in agreement with other works showing that opto-inhibition of CIN firing does not affect locomotion, anxiety‐like behavior, social memory recognition, and visuospatial object recognition (Maurice, 2015; Ztaou et al., 2018). In contrast, the behavior of PD mice, which perform poorly in all these tests, is improved by CIN silencing (Maurice, 2015). Restoring the balance between the striatofugal pathways at the level of the substantia nigra pars reticulata might be one component of the positive effect of CIN inhibition in parkinsonian condition (Maurice, 2015).

What conclusions can we draw from this brief overview? Obviously, more work is needed to understand how CIN inhibition shapes striatal output and modulates basal ganglia-related behavior. The conflicting effects of CIN firing inhibition on MSN activity and the lack of clear behavioral response in normal mice can be interpreted in different ways: (i) the light parameters (i.e. light duration, pattern or timing delivery) used to manipulate CIN firing are not physiologically relevant, (ii) CINs do not impact basal ganglia-related behaviors in physiological conditions and/or (iii) the behavioral tasks used are not appropriate to reveal CIN functions in rodents. What we know about the function of CINs comes from studies carried out in primates, describing the correlative changes in electrical activity of presumed-CINs during behavior. Optogenetics, mainly applied in rodents for technical reasons, is perfectly suited to go beyond correlational analysis and to investigate the causal implication of CINs in behavior. However, we first need to accurately describe the firing properties of these cells in rodents to be able to manipulate their activity in an appropriate way.

## Activity of Cholinergic Interneurons During Behavior

The identification of CINs in behaving animals is usually based on their unique *in vivo* extracellular firing activity (i.e. tonically active at 5 spikes/s, sometimes in a burst mode) and broad spike waveform (i.e. spike duration > 2 ms). These electrical properties are easily distinguishable from all the other striatal cell populations and represent a good signature of CINs as confirmed later by *in vivo* juxtacellular labelling (Inokawa et al., 2010; Sharott et al., 2012). Using these classification criteria, early studies first defined the pattern of CINs activity during classical conditioning. In these experiments, animals have to learn the association between a neutral stimulus (i.e. often a tone) and an unexpected reward. In this context, CINs classically respond with a pause in firing that occurs shortly after the conditioned stimulus and lasts around 200 ms. This pause can also be preceded and/or followed by excitatory burst responses (Kimura et al., 1984; Aosaki, 1994; Apicella, 2017). Interestingly, this stereotypical pause appears during learning (Aosaki, 1994) and is time-locked to the response of nigral dopaminergic neurons (Morris et al., 2004). It is also dependent on the integrity of both dopaminergic neurons (Aosaki et al., 1994; Raz et al., 1996) and glutamatergic inputs coming from the intralaminar thalamus (Matsumoto et al., 2001).

What is not yet clear is whether the pause and burst components carry different signals used to underlie specific functions (Apicella, 2002; Apicella, 2007). Importantly, these activity patterns are mostly synchronized in the CINs population (Raz et al., 1996) such that they might efficiently translate into global change of striatal ACh level, providing a temporal window for complex pre- and post-synaptic modifications of striatal network and plastic changes (Deffains and Bergman, 2015; Cox and Witten, 2019). As a consequence, the pause response of CINs is considered as a key cellular substrate for reward-based learning, and may be particularly important for stimulus-response and action-outcome associations. The exact cellular and network explanations underlying the generation of the pause/burst firing responses are not known precisely. Multiple mechanisms have been proposed to generate these responses. They all have in common the capacity to broadcast efficiently the information to spatially-distributed CINs (Goldberg and Reynolds, 2011; Schulz and Reynolds, 2013; Zhang and Cragg, 2017). Such broadcast mechanisms include:

- a change in intrinsic excitability driven by excitatory synaptic inputs (Oswald et al., 2009; Ding et al., 2010; Doig et al., 2014; Zhang et al., 2018; Reynolds et al., 2004). This scenario has been well described for cortical and intralaminar nucleus thalamic inputs but whether it can occur from any other known glutamatergic sources [such as the one coming from the pedonculopontine nucleus (Assous et al., 2019)] remained to be addressed.
- a putative effect of DA directly onto CINs (Yan et al., 1997; Maurice, 2004; Yan and Surmeier, 1997).
- a cholinergic input coming from the pedunculopontine and laterodorsal tegmental nuclei that synapse preferentially with CINs and give rise to excitatory responses (Dautan, 2014; Dautan, 2018).
- a direct inhibitory inputs coming from striatal GABAergic interneurons surrounding MSNs (Gonzales et al., 2013). Activation of one CIN is, for example, able to inhibit the firing of nearby CINs *via* nicotinic excitation of striatal GABAergic interneurons. This microcircuit allows a widespread inhibition of CINs by recurrent inhibition (Sullivan et al., 2008; Faust et al., 2016).

Also, external sources such as GABAergic neurons from the midbrain, or from the globus pallidus (GP), or from unknown origin might also synchronize CIN population (Zhang and Cragg, 2017). Among these GABAergic sources, the inhibitory inputs coming from GP neurons appear to be functionally efficient at inducing a pause in CINs (Klug et al., 2018). However, it is important to mention that the pallido-striatal inputs could originate from two main populations of GP neurons, namely the prototypic and the arkypallidal neurons (Mallet, 2012), and that the respective contribution of arkypallidal or prototypic neurons in the CIN pause response have not yet been assessed. That being said, anatomical evidences would argue that arkypallidal neurons represent a good cellular substrate to generate a synchronized pause in CIN firing. Indeed, arkypallidal neurons provide widespread striatal GABAergic inhibition (Mallet, 2012; Mallet, 2016) that densely target, with “basket-like” perisomatic contacts, the soma and proximal dendrites of CINs (Mallet, 2012). It should also be noted that CINs represent preferential targets for arkypallidal neurons, as suggested by the larger number of apposition that a single-labeled arkypallidal cells make onto CINs (Mallet, 2012). Altogether, we propose that GABAergic arkypallidal neurons constitute a powerful mechanism to generate synchronized inhibitory responses in CINs population. Whether this arkypallidal-CINs circuit is part of a feed-back or a feed-forward loop is not known but should be addressed in future studies.

Apart from the classical conditioning experiments, the contribution of CIN activity has also been tested during operant tasks. In these experiments, the animal has to execute an action to obtain a reward. These studies have revealed the involvement of CINs in more complex behavioral aspects such as contextual (Apicella, 2007), temporal (Morris et al., 2004), goal-directed action (Bradfield et al., 2013), sensori-motor gating (Ding et al., 2010), movement control/modulation (Yarom and Cohen, 2011; Nougaret and Ravel, 2015; Lee et al., 2006), and action inhibition (Lee et al., 2006). Interestingly, the expression of the pause in CIN firing is largely dependent on the behavioral task paradigm (Benhamou et al., 2014). This might actually explain some of the discrepancies originally found between monkey and rodent recordings.

In addition, recent works have taken advantage of transgenic ChAT-Cre mice to genetically identify CINs and record their activity with two-photon calcium imaging and fiber photometry, during spontaneous locomotion in head-fixed animals (Gritton, 2019; Howe, 2019; Rehani, 2019). In doing so, novel features of CIN contributions to global locomotion control have been described. In particular, one study found that CINs increase their activity during behavioral state transition, and could thus favor the transition from one behavioral state to another (Howe, 2019). Alternatively, CINs activation can reduce ongoing movement while synchronizing the activity of MSNs (Gritton, 2019). This synchronizing effect on striatal neurons is a remarkable feature especially considering that excessive expression of synchronized oscillatory activity in the beta frequency band (12–30 Hz) is a hallmark associated with PD and possibly linked to akinesia/bradykinesia in PD patients (Brown, 2007) [but see (Nambu et al., 2015; McGregor and Nelson, 2019)]. This further adds to the view that CIN dysfunctional activity contributes to the pathophysiology of PD. Indeed, there is good evidence to suggest that the loss of DA in the striatum modifies the cholinergic signalling (Tanimura, 2018; McKinley, 2019; Ztaou and Amalric, 2019) and increases the correlated activity between CINs (Raz, 2001). Although the minimal neuronal circuit generating the parkinsonian beta synchronizations in basal ganglia circuits are not known, it is possible that CIN activity represents a good candidate to promote synchronized activity in these neuronal networks. Indeed, cholinergic agonist infusion in the striatum (McCarthy, 2011) and optogenetic excitation of CINs (Kondabolu, 2016) can induce an increase in the expression of beta oscillations. In addition, CINs opto-excitation in normal animals generates parkinsonian-like motor deficits (Kondabolu, 2016) while CINs opto-inhibition in PD mice decreases motor symptoms (Maurice, 2015).

## Exploring Cholinergic Interneurons Functions in Basal Ganglia Network: Contribution of Computational Modeling

CINs modulate striatal activity during behavior. A theoretical study of the putative function of these neurons in motor learning and their possible role in pathophysiology through modeling could drive experimentally testable predictions and thereby guide further experimental investigation. Previous modeling efforts involving CINs remain relatively sparse. They range from simulating intracellular and ion-channel dynamics linked to cholinergic signaling to the effect of CIN activity modulation on behavior.

On the microscopic scale, two modeling studies have highlighted the tight coupling between DA neurons and CINs due to the dopaminergic modulation of both the intrinsic currents generating tonic firing (Aosaki et al., 1998; Maurice, 2004; Wilson and Goldberg, 2006; Deng et al., 2007) and the external inputs to CINs (Nicola et al., 2000; Pisani et al., 2000). Szalisznyó and Müller (2009) analyzed conductance-based changes in CIN subthreshold oscillations induced by DA and predicted that DA can switch CINs between stable oscillatory and fixed-point behaviors, with opposing effects of D1- and D2-type dopamine receptors. Tan and Bullock (2008) have shown that DA inputs robustly cooperate with thalamic inputs to control cue-dependent CIN pauses. Thereby, DA strongly affects performance- and learning-related dynamics in the striatum. The DA-CINs coupling could explain the adaptively scaled DA burst and the CIN burst and pause observed experimentally in response to reward-predicting cues. These changes would thus not necessarily require a modification in the weight of synapses onto CINs.

On the macroscopic scale, the influence of CINs on behavior can be either immediate, due to the modulation of striatal output by CINs, or delayed/persistent, due to ACh-dependent plasticity in the striatal network that leads to long-term changes in striatal response to its external inputs. A recent study by Vogt and Hofmann (2012) modeled the modulation in the activity of DA neurons and CINs in relation to external reward delivery and its internal expectation. They show that activity changes and their effect on learning outcome can be explained by a direct effect of neuromodulators (DA and ACh) on postsynaptic activity, even with unmodulated, two-factor spike timing-dependent plasticity (STDP). Obviously, it does not prohibit joint operation together with three-factor STDP rules. Interestingly, CIN pause could represent a time window to gate phasic DA release and “bracket” the plasticity window, while DA variations reciprocally modulate the CIN pause duration to adjust this window (Kim, 2019). In the context of reward-based motor adaptation, phasic DA release could thereby deliver reward information for reinforcement learning in a timely manner. Changes in CIN-DA interactions due to DA depletion would then produce poor performance of motor adaptation. Alternatively, CINs could act mainly on MSNs to suppress their firing and regulate local inhibitory network (Ashby and Crossley, 2011; Franklin and Frank, 2015).

To go beyond this current state of theoretical investigations, one may ask the following questions. What are the respective/specific roles of DA and ACh during learning? How redundant are these signals? Are they separable in time or space? What is the specific motor impairment expected due to the abnormal CIN activity following DA depletion and could some PD symptoms be linked to CIN signaling dysfunction? These questions may be answered by integrating current experimental evidence and DA-ACh interactions and its effect on striatal dynamics revealed by previous theoretical work in a circuit model of the basal ganglia-thalamo-cortical loop. This model may display action selection properties and DA-driven reinforcement learning (Leblois et al., 2006; Guthrie et al., 2013), as well as PD-related dysfunction under DA depletion. The computational advantages brought by CINs and the neural mechanisms can be investigated in such a theoretical framework. Specific predictions can then be derived from model concerning the effects of manipulating CIN activity in a reinforcement learning protocol. These predictions could eventually be tested experimentally with physiological recordings performed in an operant conditioning task to ensure that the suggested mechanisms are indeed at play in the striatum during motor learning.

## Concluding Remarks

Our current understanding of the role of CINs in striatal function derives mostly from extracellular recordings of TANs in monkeys. Because these neurons transiently respond to motivationally relevant cues with brief pauses, flanked by bursts of increased activity, they are classically viewed as key players in reward related learning. However, how CINs, and particularly the pause in their tonic firing, modulate striatal output has yet to be demonstrated. It is also undisputable that CINs play a key role in relation to dysfunctional aspect of basal ganglia information processing such as in PD and it seems important that future works keep dissecting the causal role of CINs in striatal circuits.

## Author Contributions

NMal, AL, NMau, and CB drafted, provided critical revision of the article, wrote, and approved the final version of the review.

## Conflict of Interest

The authors declare that the research was conducted in the absence of any commercial or financial relationships that could be construed as a potential conflict of interest.
